# Dietary Antioxidant Indices in Relation to All-Cause and Cause-Specific Mortality Among Adults With Diabetes: A Prospective Cohort Study

**DOI:** 10.3389/fnut.2022.849727

**Published:** 2022-05-04

**Authors:** Wenjie Wang, Xiaoyan Wang, Shiling Cao, Yiting Duan, Chengquan Xu, Da Gan, Wei He

**Affiliations:** ^1^Chronic Disease Research Institute, The Children's Hospital, and National Clinical Research Center for Child Health, School of Public Health, School of Medicine, Zhejiang University, Hangzhou, China; ^2^Department of Nutrition and Food Hygiene, School of Public Health, School of Medicine, Zhejiang University, Hangzhou, China

**Keywords:** dietary antioxidants indices, diabetes, mortality, NAHNES, CVD morality

## Abstract

**Background:**

The potential beneficial effect of individual antioxidants on mortality has been reported. However, the association of overall intakes of dietary antioxidants with all-cause and cause-specific mortality among adults with diabetes remained unclear.

**Methods:**

A total of 4,699 US adults with diabetes were enrolled in 2003–2014 in the National Health and Nutrition Examination Survey (NHANES) and followed for mortality until 31 December 2015. The Dietary Antioxidant Quality Score (DAQS) and the Dietary Antioxidant Index (DAI), which indicate the total antioxidant properties, were calculated based on the intakes of vitamins A, C, E, zinc, selenium, and magnesium. The Cox proportional hazards regression models were used to investigate the associations of the DAQS or the DAI with all-cause and cause-specific mortality.

**Results:**

A total of 913 deaths occurred during 27,735 person-years of follow-up, including 215 deaths due to cardiovascular disease (CVD) and 173 deaths due to cancer. The higher intakes of antioxidant vitamins A, E, magnesium, and selenium were associated with lower all-cause mortality. The adjusted hazard ratios (HRs) (95% CIs) comparing the highest DAQS (5–6) to the lowest DAQS (0–2) were 0.70 (0.53–0.92) for all-cause mortality, 0.56 (0.35–0.90) for CVD mortality, and 0.59 (0.33–1.04) for cancer mortality. Consistent inverse associations were found between the DAI and mortality.

**Conclusion:**

Higher intake of overall dietary antioxidants was associated with lower risk of death from all-cause and CVD in adults with diabetes. Future dietary intervention studies are needed to determine whether increasing overall antioxidant micronutrients intake could prevent premature death among adults with diabetes.

## Introduction

Diabetes affects 476.0 million individuals worldwide (1), which poses an extensive threat to long-term well-being. In 2019, an estimated 4.2 million adults died from diabetes globally (2). Specifically, diabetes increases risk of all-cause mortality by 2- to 3-fold and shortens life expectancy by almost 20 years (3, 4). Consistently, it is estimated that about half of deaths among people with diabetes can be attributed to cardiovascular disease (CVD) (5). Diet and nutrition have been considered as the important determinants of death, especially for adults with diabetes. Among adults with type 2 diabetes, more than 300,000 deaths can be attributable to dietary risks annually in the world (6).

Oxidative stress is a result of an imbalance between prooxidants and antioxidants in the body and has been proven to be a risk phenomenon in diabetes and death. Although harmful, oxidants are critical for many physiological responses in cells at appropriate concentrations, including intracellular signaling and redox regulation. Oxidants are signaling molecules that regulate cell proliferation, apoptosis, and gene expression at low levels (7, 8). Simultaneously, moderate amounts of reactive oxygen species are essential mediators of protective apoptosis and phagocytosis in cancer (9). Dietary antioxidants, due to their ability to remove free radicals and inhibit lipid peroxidation, have received extensive attention in the recent decades. Antioxidants are molecules that slow or prevent other molecules from oxidizing. Researchers have identified the potential beneficial effects of antioxidants in terminating chain reactions by scavenging free radical intermediates and inhibiting other oxidative reactions (10).

Previous clinical trials and cohort studies found that the higher intake of antioxidants improved glycemic control indicators and was associated with lower risk of mortality (11–16). These studies tended to be conducted in the general population and mainly focused on the influence of single antioxidants. However, the impact of food on health is influenced not only by individual nutrients, but also by their interactions. Until now, limited evidence is available on the association between the overall dietary antioxidants intake and mortality among adults with diabetes. Therefore, this study aimed to investigate the associations of overall dietary antioxidants intake, indicated by the Dietary Antioxidant Quality Score (DAQS) and the Dietary Antioxidant Index (DAI), with the risk of mortality from all-cause, cardiovascular disease (CVD), and cancer in adults with diabetes, using data from the National Health and Nutrition Examination Survey (NHANES) (2003–2014).

## Materials and Methods

### Study Population

The National Health and Nutrition Examination Survey (NHANES) is a cross-sectional survey that uses a complex, multistage, and stratified probability sampling method to obtain nationally representative health and nutrition data of the noninstitutionalized US population. Participants' data were collected from household interviews and laboratory examinations. More details of the NHANES have previously been illustrated elsewhere (17).

This investigation analyzed data from 6 2-year cycles (2003–2004, 2005–2006, 2007–2008, 2009–2010, 2011–2012, and 2013–2014). Individuals (aged ≥18 years) with diabetes were included in this study. Diabetes was defined as self-reported doctor diagnosis (ever been told by a clinical consultant or health professional that you have diabetes or sugar diabetes), taking insulin and oral hypoglycemic agents, fasting plasma glucose level ≥ 7.0 mmol/l, and/or glycated hemoglobinA_1c_ (HbA_1c_) level ≥ 6.5% (18). We then further excluded participants with missing or unknown data on mortality and dietary antioxidants intake and females who were pregnant, leaving a total of 4,699 adults with diabetes for the final study (Supplementary Figure 1).

The NHANES protocol was approved by the National Center for Health Statistics Research Ethics Review Board and all the participants provided informed consent.

### Dietary Assessment

The NHANES participants' food and nutrients intake were evaluated by using nonconsecutive 2-day 24-h dietary recall performed by trained interviewers. The first dietary recall was conducted in-person in the Mobile Examination Center and the second dietary recall was collected through a telephone interview approximately 3–10 days later. The Automated Multiple-Pass Method (AMPM) was employed to record the specific consumption of all the foods and beverages in the past day. Dietary antioxidants micronutrients and total energy intake values were calculated using the United States Department of Agriculture (USDA) Food and Nutrient Database for Dietary Studies (FNDDS) (19). Questionnaire interview was utilized to obtain information on dietary supplements usage in the past 30 days, including the frequency, duration, and dose intake of dietary supplements (20).

### Calculation of the Dietary Antioxidant Quality Score and the Dietary Antioxidant Index

The DAQS and the DAI were calculated based on six dietary antioxidant micronutrients, including vitamins A, C, E, zinc, magnesium, and selenium. For the DAQS, we compared each of the above six nutrients/minerals intake to their respective daily recommended intake (RDI) for US adults released by the Dietary Guidelines for Americans 2015–2020 (21). For each antioxidant vitamin/mineral, the DAQ scores of 0 and 1 were defined as intake <2/3 of the RDI and the intake ≥2/3 of the RDI, respectively, according to Rivas and colleagues' method (22). The summed DAQS ranged from 0 (poor quality) to 6 (high quality). The DAQS was then classified into the three groups: 1–2 (low quality), 3–4 (medium quality), and 5–6 (high quality).

The DAI was calculated by using the method proposed by Wright et al. in the former study (23). We standardized each of the above antioxidant micronutrients by subtracting the mean and dividing by the SD to estimate the DAI. Then, we add up the standardized intakes of these micronutrients with equal weight to calculate the composite DAI, as shown below:


$$\begin{array}{l} \text{DAI }=\;\overset{\text{n }=\;6}{\underset{\text{i }=\;1}{\sum }}\frac{\text{Individual Intake }-\text{ Mean}}{\text{SD}} \end{array}$$

### Evaluation of Dietary Total Antioxidant Capacity

Each antioxidant had a different antioxidant capacity; the antioxidant capacity of each antioxidant was determined using 2,2'-azino-bis(3-ethylbenzothiazoline-6-sulfonic acid) (ABTS) and expressed as vitamin C equivalent (VCE) (24, 25). Daily antioxidant intake was multiplied by the antioxidant capacity (VCE) of each antioxidant and added together to produce total antioxidant capacity (TAC).

### Ascertainment of Mortality

Mortality outcomes and follow-up time of the NHANES participants were identified by the NHANES-linked National Death Index public access files. The NHNAES-linked mortality data have been used in a large number of publications (26–28). Follow-up time was defined from the date of participation to the date of death on 31 December 2015, whichever came first. The International Statistical Classification of Disease, 10th Revision (ICD-10) was employed to determine death from CVD (I00–I09, I11, I13, I20–I51, and I60–I69) and death from cancer death (C00–C97) (29).

### Assessment of Confounding Factors

The potential confounding factors in this study were collected from household interviews using standardized questionnaires, including age (years), gender (male/female), race/ethnicity (non-Hispanic white/non-Hispanic black/Mexican American/other), body mass index (BMI) (kg/m^2^), education level (less than high school/high school or equivalent/college or above), income (< $20,000/$20,000–$75,000/>$75,000), smoking status (never smoker/former smoker/current smoker <15 cigarettes/day/current smoker ≥15 cigarettes/day), exercise regularly (yes/no), drinking currently (yes/no), total energy intake (kcal/day), dietary supplements use (yes/no), self-reported chronic noncommunicable diseases (NCDs), including hypertension, dyslipidemia, heart diseases, and cancer (yes/no), family history of diabetes (yes/no), medication use for diabetes (insulin/diabetic pills/none), duration of diabetes (years), and HbA_1c_ (%).

### Statistical Analysis

Sample weight clustering and stratification were incorporated across all the analyses to illustrate the complex survey design because of unequal sampling selection probability and oversampling of certain subgroups. Selected baseline characteristics of participants were presented as means (SE) or *n* (percentages), stratified by the three categories of the DAQS or the DAI. The levels of significances were examined by using general linear regression (continuous variables) and the chi-squared test (categorical variables).

The Cox proportional hazards regression models were employed to evaluate the hazard ratios (HRs) and 95% CIs for the association of antioxidant (DAQS, DAI, and their components) with mortality. Model 1 was adjusted for age, gender, and race. Model 2 was further adjusted for BMI, education, income, exercise, smoking, drinking, dietary supplements, and total energy intake. Model 3 was additionally adjusted for hypertension, dyslipidemia, heart diseases, cancer, family history of diabetes, medication use for diabetes, duration of diabetes, and HbA_1c_. Tests for linear trends were performed by assigning the medium value of each group as continuous variables. Moreover, we performed subgroup analyses in accordance with some stratified variables. *P* for interaction was assessed from multivariate-adjusted model by using multiplicative terms between the DAQS or the DAI (continuous) and stratification factors (dichotomous).

We performed a series of sensitivity analyses to test the robustness of our results. First, given that certain dietary covariates and diet quality were generally controlled in previous cohort studies regarding the relationship between single antioxidants and mortality (30, 31), we additionally adjusted for these nutrients in our final model. Second, we reanalyzed the data after excluding the participants who follow-up times <2 years and participants who taken antioxidant supplements to investigate the potential effect of dietary supplements. Third, we adjusted for blood lipids, C-reactive protein (CRP), Homeostatic Model Assessment for Insulin Resistance (HOMA-IR), and HOMA-β function index in the final model to examine the potential mechanisms. Fourth, considering the sex difference of antioxidants intake, the associations between the DAQS and the DAI and mortality were estimated separately among males and females. Finally, we estimated the association between dietary total antioxidant capacity (TAC), another measure of total dietary antioxidant intake, and mortality among people with diabetes.

All the statistical tests were performed in R project 3.5.3 (The R Foundation for Statistical Computing, Vienna, Austria). Two-sided *P*-value <0.05 was considered to be statistically significant.

## Results

### Participant's Characteristics

A total of 913 deaths were identified out of the 4,699 diabetes patients, including 215 deaths due to CVD and 173 deaths due to cancer. Table 1 presents the baseline characteristics of study participants by the DAQS and the DAI. The participants with the higher DAQS and DAI were more likely to be non-Hispanic white, regular exercisers, and current drinkers; were less likely to be current smokers; and had higher education, income levels, and total energy intake.

**Table 1:** Baseline characteristics by the Dietary Antioxidant Quality Score (DAQS) and the Dietary Antioxidant Index (DAI) among adults with diabetes in the National Health and Nutrition Examination Survey (2003–2014).

	**Dietary Antioxidant Quality Score (DAQs)**				**Dietary Antioxidant Index (DAI)**			
	**0–2**	**3–4**	**5–6**	***P-*value^a^**	**Tertile 1**	**Tertile 2**	**Tertile 3**	***P-*value^a^**
	***N =* 1,412**	***N =* 1,984**	***N =* 1,307**		***N =* 1,568**	***N =* 1,569**	***N =* 1,566**	
Age, year	59.70 (0.43)	58.84 (0.42)	58.89 (0.45)	0.182	60.72 (0.39)	59.77 (0.50)	57.23 (0.45)	<0.001
Female, %	619 (45.9)	1,006 (50.8)	663 (49.7)	0.166	955 (66.2)	813 (52.9)	820 (53.0)	0.111
Race/ethnicity, %				<0.001				<0.001
Non-hispanic white	479 (55.5)	732 (61.2)	582 (69.6)		513 (53.5)	622 (64.8)	658 (66.9)	
Non-hispanic black	453 (21.0)	513 (15.6)	318 (12.7)		514 (22.7)	396 (14.4)	374 (12.6)	
Mexican American	246 (8.5)	424 (10.6)	232 (8.0)		293 (9.1)	305 (9.3)	304 (9.2)	
Others	234 (15.0)	315 (12.6)	175 (9.7)		248 (14.7)	246 (11.5)	230 (11.3)	
Education level, %				<0.001				<0.001
<11th grade	660 (35.8)	757 (27.5)	352 (17.7)		754 (37.8)	588 (26.2)	427 (18.4)	
High school Grad/GAD or equivalent	329 (25.5)	479 (27.5)	301 (24.0)		370 (26.8)	372 (26.2)	367 (24.9)	
College or above	423 (38.7)	748 (45.0)	670 (58.3)		444 (35.3)	609 (47.7)	772 (56.7)	
Income, %				<0.001				<0.001
Under $20,000	475 (26.9)	549 (20.6)	306 (16.5)		556 (29.8)	437 (20.6)	337 (14.6)	
$20,000–$75,000	711 (53.1)	1,062 (55.7)	692 (54.3)		777 (52.7)	840 (55.3)	848 (55.3)	
Over $75,000	217 (20.0)	373 (23.7)	309 (29.2)		235 (17.5)	292 (24.1)	381 (30.1)	
Smoking status, %				<0.001				0.001
Never smoker	581 (41.4)	912 (46.8)	635 (48.7)		726 (46.8)	708 (45.7)	694 (47.7)	
Former smoker	474 (32.2)	673 (35.3)	456 (38.0)		476 (29.0)	562 (36.5)	562 (37.0)	
Current smoker <15 cigarettes/day	179 (13.6)	186 (8.7)	100 (6.7)		185 (12.3)	137 (8.4)	143 (7.0)	
Current smoker >15 cigarettes/day	124 (12.8)	142 (9.2)	79 (6.6)		120 (11.9)	105 (9.4)	120 (8.4)	
Drinking currently, %	808 (58.9)	1,161 (61.4)	818 (67.4)	0.008	817 (53.6)	902 (60.2)	1,068 (71.6)	<0.001
Exercise regularly, %	186 (14.8)	324 (18.5)	250 (22.2)	0.004	214 (15.7)	257 (19.6)	289 (20.2)	0.005
BMI, kg/m^2^	32.49 (0.24)	32.98 (0.27)	33.25 (0.30)	0.058	32.51 (0.22)	33.01 (0.31)	33.20 (0.31)	0.060
Total energy, kcal/day	1359.31 (20.51)	1,885.62 (22.10)	2,384.44 (33.10)	<0.001	1,246.22 (13.79)	1,765.42 (14.66)	2,532.38 (25.51)	<0.001
Dietary supplements use, %	590 (43.4)	997 (54.0)	793 (63.3)	<0.001	698 (48.6)	812 (53,5)	870 (59.0)	0.001
**Ever told you had**								
Hypertension, %	964 (67.1)	1,291 (64.6)	860 (64.3)	0.652	1,102 (69.7)	1,021 (63.6)	992 (63.1)	0.018
Dyslipidemia,%	780 (57.8)	1,070 (56.1)	745 (59.6)	0.099	868 (56.3)	860 (57.9)	867 (58.5)	0.263
Heart diseases, %	450 (31.2)	511 (24.6)	299 (21.4)	<0.001	483 (31.4)	424 (25.7)	353 (20.2)	<0.001
Cancer, %	196 (15.7)	265 (14.7)	196 (17.4)	0.577	218 (15.2)	225 (16.4)	214 (15.8)	0.397
Family history of diabetes, %	303 (24.8)	458 (25.6)	294 (24.1)	0.331	379 (27.4)	325 (23.8)	351 (24.0)	0.121
Duration of diabetes, years	11.29 (0.39)	10.64 (0.27)	10.07 (0.30)	0.014	11.56 (0.35)	10.52 (0.23)	10.02 (0.28)	<0.001
Glycohemoglobin, %	7.23 (0.06)	7.25 (0.05)	7.15 (0.06)	0.288	7.20 (0.05)	7.17 (0.05)	7.26 (0.06)	0.319

*Values are given as weighted mean (standard error) for continuous variables and number (weighted percentages) for categorical variables*.

a *P-value was calculated by linear model for continuous variables and chi square test for categorical variables*.

#### Associations of Individual Antioxidant Micronutrients With Mortality

**Figure 1:**
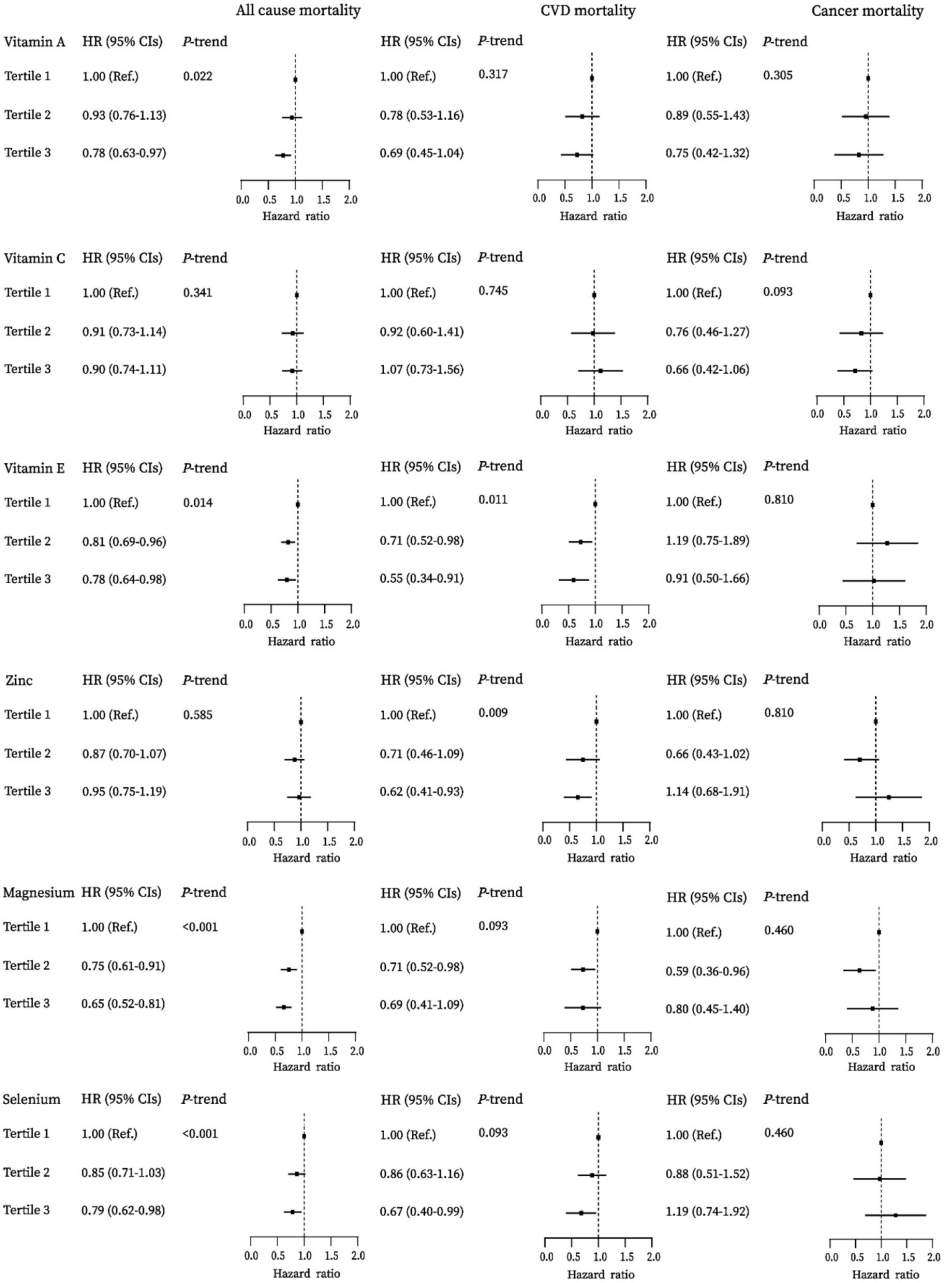
Associations between vitamins A, C, E, zinc, magnesium, and selenium with all-cause, cardiovascular disease (CVD), and cancer mortality among diabetes in the National Health and Nutrition Examination Survey (NHANES) (2003–2014).

Figure 1 presents the association of the component antioxidant of the DAQS and the DAI with mortality among adults with diabetes. Higher antioxidants intakes were inversely associated with all-cause mortality. The adjusted hazard ratios (HRs) (95% CIs) comparing the highest tertile to the lowest tertile were 0.78 (0.63–0.97) for vitamin A, 0.78 (0.64–0.98) for vitamin E, 0.65 (0.52–0.81) for magnesium, and 0.79 (0.62–0.98) for selenium, respectively (all *P*_trend_ <0.05). Higher intakes of vitamin E, zinc, and selenium were associated with lower risk of CVD mortality; the HRs (95% CIs) for the highest vs. lowest tertiles were 0.55 (0.34–0.91), 0.62 (0.41–0.93), and 0.67 (0.40–0.99), respectively (all *P*_trend_ <0.05). No significant association of these antioxidants with cancer mortality was observed in this study (Supplementary Table 1).

#### Associations of Overall Antioxidants Intake With Mortality

Table 2 shows the association of overall antioxidants intake with mortality. In the multivariate-adjusted models, the higher DAQS was significantly associated with decreased risk of all-cause and CVD mortality. HRs (95% CIs) from the lowest to highest DAQS categories were 1.00, 0.77 (0.63–0.94), and 0.70 (0.53–0.92) (*P*_trend_ = 0.003) for all-cause mortality and 1.00, 0.75 (0.50–1.13), and 0.56 (0.35–0.90) (*P*_trend_ = 0.020) for CVD mortality. Consistent result was discovered on the association between the DAI and mortality and the HRs (95% CIs) from first to third tertiles of the DAI were 1.00, 0.76 (0.63–0.92), and 0.73 (0.55–0.96) (*P*_trend_ = 0.014) for all-cause mortality and 1.00, 0.74 (0.51–1.06), and 0.51 (0.31–0.82) (*P*_trend_ = 0.005) for CVD mortality. The obvious association of overall antioxidants intake with cancer mortality was not found among adults with diabetes in this study.

**Table 2:** Association of the Dietary Antioxidant Quality Score (DAQS) and the Dietary Antioxidant Index (DAI) with all-cause and cause-specific mortality among adults with diabetes in the National Health and Nutrition Examination Survey (2003–2014).

	**Dietary Antioxidant Quality Score (DAQs)**			***P*-trend^a^**
	**0–2**	**3–4**	**5–6**	
All-cause mortality (case/*n*)	329/1,412	376/1,984	208/1,307	
Model 1	1.00	0.75 (0.61–0.90)	0.60 (0.49–0.74)	<0.001
Model 2	1.00	0.78 (0.64–0.95)	0.66 (0.54–0.82)	<0.001
Model 3	1.00	0.77 (0.63–0.94)	0.70 (0.53–0.92)	0.003
CVD mortality (case/*n*)	77/1,412	95/1,984	43/1,307	
Model 1	1.00	0.75 (0.49–1.14)	0.48 (0.30–0.78)	0.004
Model 2	1.00	0.79 (0.52–1.19)	0.55 (0.34–0.89)	0.016
Model 3	1.00	0.75 (0.50–1.13)	0.56 (0.35–0.90)	0.020
Cancer mortality (case/*n*)	66/1,412	69/1,984	38/1,307	
Model 1	1.00	0.76 (0.50–1.17)	0.69 (0.43–1.11)	0.121
Model 2	1.00	0.80 (0.53–1.22)	0.75 (0.46–1.23)	0.251
Model 3	1.00	0.72 (0.45–1.17)	0.59 (0.33–1.04)	0.062
	**Dietary Antioxidant Index (DAI)**			
	**Tertile 1**	**Tertile 2**	**Tertile 3**	
All-cause mortality (case/*n*)	370/1,568	301/1,569	242/1,566	
Model 1	1.00	0.72 (0.59–0.87)	0.61 (0.52–0.73)	<0.001
Model 2	1.00	0.75 (0.62–0.92)	0.68 (0.57–0.81)	<0.001
Model 3	1.00	0.76 (0.63–0.92)	0.73 (0.55–0.96)	0.014
CVD mortality (case/*n*)	86/1,568	78/1,569	51/1,566	
Model 1	1.00	0.70 (0.48–1.02)	0.42 (0.26–0.68)	<0.0011
Model 2	1.00	0.75 (0.52–1.09)	0.48 (0.30–0.78)	0.002
Model 3	1.00	0.74 (0.51–1.06)	0.51 (0.31–0.82)	0.005
Cancer mortality (case/*n*)	69/1,568	54/1,569	50/1,566	
Model 1	1.00	0.78 (0.49–1.25)	0.53 (0.53–1.33)	0.481
Model 2	1.00	0.80 (0.50–1.27)	0.91 (0.56–1.48)	0.738
Model 3	1.00	0.73 (0.44–1.21)	0.73 (0.35–1.53)	0.397

*Model 1, adjusted for age, sex and race/ethnicity. Model 2, model 1 + body mass index, smoking status, drinking currently, education level, income level, exercise regularly. Model 3, model 2 + total energy intake, dietary supplements use, self-reported hypertension, dyslipidemia, heart disease, cancer, family history of diabetes, medication use for diabetes (insulin/diabetic pills/none), duration of diabetes and hemoglobin A_1c_*.

a *Calculated by using the median value for each DAQs or DAI category as a continuous variable*.

### Subgroup and Sensitivity Analyses

Table 3 shows stratified analyses by the patients' characteristics. In almost all the subgroups, patients with the higher DAQS were more likely to have lower all-cause mortality. This was particularly evident among older adults aged > 60 years, whites, smokers, inactive patients, patients without family history of diabetes, and those with comorbidities (all *P*_interaction_ <0.05). Moreover, the inverse association between the DAI and all-cause mortality was stronger among older, smokers, and those with comorbidities (all *P*_interaction_ < 0.05).

**Table 3:** Association of the Dietary Antioxidant Quality Score (DAQS) or the Dietary Antioxidant Index (DAI) with all-cause mortality among adults with diabetes in the National Health and Nutrition Examination Survey (2003–2014), stratified by selected patients' characteristics^a^.

	**Dietary Antioxidant Quality Score (DAQs)**			***P*-trend**	***P* interaction^b^**
	**0–2**	**3–4**	**5–6**		
**Age**					0.002
≤ 60	1.00	0.91 (0.62–1.33)	0.84 (0.53–1.33)	0.528	
>60	1.00	0.71 (0.57–0.89)	0.60 (0.45–0.80)	0.002	
**Race/ethnicity**					0.004
Non-hispanic white	1.00	0.66 (0.51–0.85)	0.59 (0.42–0.83)	0.001	
Non-white	1.00	0.96 (0.79–1.43)	0.95 (0.77–1.45)	0.710	
**BMI**					0.067
<30	1.00	0.79 (0.62–0.99)	0.62 (0.43–0.90)	0.015	
≥30	1.00	0.85 (0.67–1.06)	0.83 (0.62–1.13)	0.211	
**Smoking status**					0.004
Never smoker	1.00	0.82 (0.57–1.18)	0.88 (0.58–1.34)	0.558	
Ever smoker	1.00	0.77 (0.61–0.98)	0.66 (0.45–0.96)	0.022	
**Drinking currently**					0.330
Yes	1.00	0.76 (0.61–0.94)	0.69 (0.52–0.92)	0.009	
No	1.00	0.74 (0.50–1.08)	0.88 (0.60–1.29)	0.660	
**Exercise regularly**					0.016
Yes	1.00	0.69 (0.40–1.18)	0.91 (0.36–1.75)	0.263	
No	1.00	0.83 (0.67–1.03)	0.76 (0.62–0.94)	0.028	
**Family history of diabetes**					0.003
Yes	1.00	0.91 (0.68–1.22)	0.93 (0.73–1.45)	0.621	
No	1.00	0.78 (0.65–0.94)	0.60 (0.42–0.84)	0.004	
**Comorbidity** ^‡^					0.030
Yes	1.00	0.80 (0.65–0.98)	0.68 (0.55–0.86)	0.001	
No	1.00	0.78 (0.37–1.24)	0.78 (0.34–1.39)	0.059	
	**Dietary Antioxidant Index (DAI)**				
	**Tertile**	**Tertile 2**	**Tertile 3**		
**Age**					0.005
≤ 60	1.00	0.78 (0.52–1.18)	0.73 (0.48–1.11)	0.139	
>60	1.00	0.83 (0.71–0.99)	0.65 (0.42–0.91)	0.002	
**Race/ethnicity**					0.100
Non-hispanic white	1.00	0.65 (0.51–0.84)	0.60 (0.47–0.76)	<0.001	
Non-white	1.00	0.97 (0.71–1.31)	0.95 (0.64–1.38)	0.426	
**BMI**					0.063
<30	1.00	0.79 (0.63–0.99)	0.77 (0.60–0.98)	0.028	
≥30	1.00	0.85 (0.68–1.06)	0.85 (0.67–1.07)	0.150	
**Smoking status**					0.040
Never smoker	1.00	0.74 (0.53–1.05)	0.96 (0.63–1.78)	0.117	
Ever smoker	1.00	0.74 (0.59–0.94)	0.59 (0.43–0.79)	0.002	
**Drinking currently**					0.277
Yes	1.00	0.73 (0.43–1.22)	0.55 (0.30–0.97)	0.025	
No	1.00	0.73 (0.52–1.04)	1.17 (0.71–1.92)	0.593	
**Exercise regularly**					0.458
Yes	1.00	0.64 (0.34–1.23)	0.79 (0.51–1.91)	0.208	
No	1.00	0.82 (0.66–1.02)	0.77 (0.62–0.95)	0.015	
**Family history of diabetes**					0.058
Yes	1.00	0.88 (0.65–1.18)	0.93 (0.68–1.28)	0.641	
No	1.00	0.80 (0.66–0.97)	0.77 (0.62–0.95)	0.011	
**Comorbidity** ^‡^					
Yes	1.00	0.77 (0.63–0.93)	0.71 (0.53–0.95)	0.009	0.014
No	1.00	0.87 (0.41–1.85)	0.78 (0.39–1.54)	0.407	

a *Model was adjusted for age, sex, race/ethnicity, body mass index (BMI), smoking status, drinking currently, exercise, education level, income level, total energy intake, dietary supplements use, self-reported hypertension, dyslipidemia, heart disease, cancer, family history of diabetes, duration of diabetes and hemoglobin A_1c_, with exception of stratifying factors*.

b *P for interaction was assessed from multivariable adjusted model by using the cross-product term between DAQs or DAI (continuous) and stratification factors (dichotomous)*.

‡ *Self-reported comorbidities including hypertension, heart diseases, dyslipidemia, or cancer*.

Restricting the analyses to patients with > 2 years of follow-up (Supplementary Table 2), while excluding those patients who took antioxidant supplements (Supplementary Table 3), further adjusting for monounsaturated fatty acid/saturated fatty acid (MUFA/SFA) ratio, polyunsaturated fatty acids (PUFAs)/SFA ratio, dietary cholesterol, dietary fiber (Supplementary Table 4, model 2), or B vitamins intake, including vitamins B1, B2, B6, and folate (Supplementary Table 4, model 3); diet quality calculated by the Healthy Eating Index-2015 (HEI-2015) (Supplementary Table 4, model 4) provided similar results as in the main analyses. Stratified analyses by gender also yielded similar results (Supplementary Table 5). The associations were not materially altered when total antioxidant capacity (TAC), instead of absolute dietary intake, was examined as the exposures (Supplementary Table 6).

## Discussion

In this large prospective study of nationally representative US adults with diabetes, we found that higher overall antioxidants intakes, indicated by the DAQS and the DAI, were associated with lower risk of all-cause and CVD mortality. The inverse associations remained statistically significant even after adjusting for a series of conventional dietary risk factors.

To the best of our knowledge, this is the first prospective study to investigate the association of dietary overall antioxidants intake with the risk of all-cause and cause-specific mortality in adults with diabetes. Previous studies have investigated the association between dietary antioxidants and mortality in the general population. Among patients with diabetes, lower dietary antioxidants level is common owing to medication interactions (such as metformin), malnutrition, and unhealthy eating habits (32). However, evidence is limited among patients with diabetes who had a lower dietary antioxidants intake and increased risk of all-cause and CVD mortality (5, 33). This study filled this gap and suggests that adequate intake of overall antioxidant micronutrients may help to reduce mortality among adults with diabetes.

Previous studies have investigated the association between individual antioxidant micronutrients and mortality, but with inconsistent conclusions. Some studies reported an inverse association between intakes of vitamins A, C, E, zinc, selenium, and magnesium and the risk of mortality (13, 14, 34, 35), whereas others found no significant association (12, 36, 37). For instance, Enstrom et al. found that the higher vitamin C intake was inversely associated with death from all-cause and CVD in the NHANES I Epidemiologic Follow-up Study (NHEFS) among 11,348 US adults (38). However, a recent Eastern Europe cohort study of 28,945 adults aged 45–69 years found no significant association (12). All the above studies examined the relationship between single antioxidant and mortality. Over the past few decades, human nutrition science has shifted from focusing on specific nutrients to emphasizing overall dietary quality. These findings may, thus, be biased because they ignored important information about the complex correlations and interactions between these antioxidants (39). This study minimized these biases by using the overall dietary antioxidants. In this study, we prospectively found that the higher holistic intakes of antioxidant vitamins A, C, E, zinc, selenium, and magnesium were associated with lower risk of all-cause and CVD mortality. Our findings emphasized the importance of adequate overall antioxidant micronutrients intake in adults with diabetes. Furthermore, the statistically insignificant association between dietary antioxidant indices and cancer mortality possibly because of the limited number of cases and needs to be further confirmed in larger cohort investigations.

In subgroup analysis, we observed significant interactions between the dietary antioxidant indices and the predefined risk factors on mortality. Both the DAQS and the DAI showed a stronger negative association with mortality in older, smokers, and those with other comorbidities. Despite the specific reasons being unclear, our findings suggest that higher risk diabetic population may benefit more from overall dietary antioxidants intake. However, validation in other studies is needed before high-risk diabetes populations can be recommended to intake more antioxidant-rich foods such as fruits, dark-green vegetables, nuts, and seafood (40, 41). Besides, we compared the associations of overall dietary antioxidant intake with mortality among diabetics and nondiabetics, respectively. We found that the inverse association between the DAQS and the DAI and CVD mortality seemed to be more pronounced among diabetics (Supplementary Table 7), probably since these individuals have high levels of oxidative stress and an exogenous antioxidant intake appears to exerts a stronger protective effect in people with high levels of innate or acquired reactive oxygen species (ROS) (42).

Several epidemiological studies have reported that antioxidants supplementation reduced the blood insulin and lipids profile and both the DAI and the DAQS were inversely associated with four inflammation biomarkers among type 2 diabetes patients (43–45). Therefore, we additionally adjusted the blood lipids, CRP, HOMA-IR, and HOMA-β in the final model to examine the underlying mechanisms in this study. We found that the association between overall antioxidants intake and CVD mortality was attenuated to nonsignificance after further adjusting for CRP, HOMA-IR, and HOMA-β (Supplementary Table 8), suggesting that the overall antioxidants intake may exert its effect through inflammation and insulin pathways. Except for the above hypothesis, another possible mechanism would be the antagonistic effect of antioxidants on oxidative stress and lipid peroxidation. Previous animal and human studies indicated that antioxidants could be involved in inhibiting oxidative modification of low-density lipoprotein (LDL) and increasing total glutathione (GSH), thereby preventing atherosclerosis and cardiac complications (46, 47). Moreover, other studies performed among type 2 diabetic patients suggested that overall antioxidants supplementation significantly decreased the levels of urinary albumin excretion and oxidative stress, thereby improving glomerular function and endothelial dysfunction (48, 49).

Reactive oxygen species (ROS), a cellular oxidant, is constantly produced in animal and human cells. Excessive ROS can induce oxidative stress, resulting in cell damage and ultimately lead to many degenerative diseases and aging (50). Therefore, the body has an antioxidant network to remove overproduced ROS and prevent their damaging effects. However, the beneficial effects of appropriate concentrations of oxidants on cellular physiology are increasingly being demonstrated. Oxidants are essential mediators of antibacterial phagocytosis, detoxification reactions by cytochrome P450 complexes, and elimination of apoptosis in cancer cells and other life-threatening cells (42, 51). Overdose of antioxidants can lead to “antioxidant stress,” which impairs the physiological functions of oxidants and interferes with the immune system's basic defense mechanisms to fight against bacteria and clear damaged cells (9). In addition, when consumed in excess, antioxidants can act as prooxidants by increasing oxidative stress (52). Diabetic patients were reported to have a lower intake of dietary antioxidants, while a higher level of oxidative stress, which was confirmed in this study as well (Supplementary Table 9). In addition, previous studies suggested that antioxidant supplementation is indeed effective in reducing oxidative stress level when the initial oxidative stress levels are higher than the set regulatory level (53). Thus, appropriate exogenous antioxidant supplementation should be recommended for patients with diabetes to prevent the risk of premature death.

This investigation was strengthened by the prospective design, the nationally representative sample, and the high-quality data with detailed information on potential confounding factors. Moreover, the definition of diabetes is mainly based on fasting glucose, HbA_1c_ level, and diabetes medications use, in addition to self-reported diabetes, which greatly decreases the probability of misclassification. This study also has several limitations. First, although the 2 days 24-h dietary recall was the valid method to acquire the dietary intake, but the subjective recall poses a great challenge for obtaining accurate evaluation. Second, despite adjusting the covariates comprehensively, we still could not completely exclude the unmeasured confounding factors. Third, we only used data collected at baseline, but dietary habits and other exposures may change during long-term follow-up, which may contribute to the misclassification bias. Fourth, other indices such as ferric reducing antioxidant capacity (FRAP) and total radical-trapping antioxidant parameter (TRAP), which also measure the overall antioxidant potential, but could not be calculated in this study, since no FRAP and TRAP databases have been developed for US diet (54, 55). Finally, this study fails to distinguish the types of diabetes or obtain information about the severity of diabetes. Future studies are needed to examine this association in terms of type 1 and type 2 diabetes to provide more comprehensive guidance.

## Conclusion

In conclusion, we found that the higher dietary intake of overall antioxidant micronutrients was associated with lower risk of all-cause and CVD mortality among people with diabetes. Future studies are needed to determine whether dietary intervention to promote antioxidant-rich dietary patterns could prevent premature death among adults with diabetes.

## Data Availability Statement

The datasets presented in this study can be found in online repositories. The names of the repository/repositories and accession number(s) can be found below: https://www.cdc.gov/nchs/nhanes/.

## Ethics Statement

The NHANES protocols were approved by the National Center for Health Statistics (NCHS) Research Ethics Review Board.

## Author Contributions

WH contributed to the conceptualization and design of the study, supervised the data collection, statistical analyses, initial drafting of the manuscript, and reviewed and revised the manuscript. WW and XW conceptualized and designed the study, completed the statistical analyses, drafted the initial manuscript, and reviewed and revised the manuscript. SC, YD, and CX assisted with the data interpretation and reviewed and revised the manuscript. All authors have read and approved the final version of the manuscript.

## Funding

This study was supported by the Zhejiang University through Hundred Talents Program. The funders had no role in the design and conduct of the study, in the collection, analysis, and interpretation of the data, or in the preparation, review, or approval of the manuscript.

## Conflict of Interest

The authors declare that the research was conducted in the absence of any commercial or financial relationships that could be construed as a potential conflict of interest.

## Publisher's Note

All claims expressed in this article are solely those of the authors and do not necessarily represent those of their affiliated organizations, or those of the publisher, the editors and the reviewers. Any product that may be evaluated in this article, or claim that may be made by its manufacturer, is not guaranteed or endorsed by the publisher.

## References

[1] Lin X Xu Y Pan X Xu J Ding Y Sun X. Global, regional, and national burden and trend of diabetes in 195 countries and territories: an analysis from 1990 to 2025. Sci Rep. (2020) 10:14790. 10.1038/s41598-020-71908-9 32901098 PMC7478957

[2] Saeedi P Salpea P Karuranga S Petersohn I Malanda B Gregg EW. Mortality attributable to diabetes in 20-79 years old adults, 2019 estimates: results from the International Diabetes Federation Diabetes Atlas, 9(th) edition. Diabetes Res Clin Pract. (2020) 162:108086. 10.1016/j.diabres.2020.108086 32068099

[3] Bragg F Holmes MV Iona A Guo Y Du H Chen Y. Association between diabetes and cause-specific mortality in rural and urban areas of China. JAMA. (2017) 317:280–9. 10.1001/jama.2016.19720 28114552 PMC6520233

[4] Yu M Zhan X Yang Z Huang Y. Measuring the global, regional, and national burden of type 2 diabetes and the attributable risk factors in all 194 countries. J Diabetes. (2021) 13:613–39. 10.1111/1753-0407.13159 33486878

[5] Einarson TR Acs A Ludwig C Panton UH. Prevalence of cardiovascular disease in type 2 diabetes: a systematic literature review of scientific evidence from across the world in 2007-2017. Cardiovasc Diabetol. (2018) 17:83. 10.1186/s12933-018-0728-6 29884191 PMC5994068

[6] Collaborators GBDD. Health effects of dietary risks in 195 countries, 1990-2017: a systematic analysis for the global burden of disease study 2017. Lancet. (2019) 393:1958–72. 10.1016/S0140-6736(19)30041-8 30954305 PMC6899507

[7] Murrell GA Francis MJ Bromley L. Modulation of fibroblast proliferation by oxygen free radicals. Biochem J. (1990) 265:659–65. 10.1042/bj2650659 2154966 PMC1133685

[8] Schreck R Rieber P Baeuerle PA. Reactive oxygen intermediates as apparently widely used messengers in the activation of the NF-kappa B transcription factor and HIV-1. EMBO J. (1991) 10:2247–58. 10.1002/j.1460-2075.1991.tb07761.x 2065663 PMC452914

[9] Poljsak B Milisav I. The neglected significance of “antioxidative stress”. Oxid Med Cell Longev. (2012) 2012:480895. 10.1155/2012/480895 22655114 PMC3357598

[10] Genestra M. Oxyl radicals, redox-sensitive signalling cascades and antioxidants. Cell Signal. (2007) 19:1807–19. 10.1016/j.cellsig.2007.04.009 17570640

[11] Aune D Keum N Giovannucci E Fadnes LT Boffetta P Greenwood DC. Dietary intake and blood concentrations of antioxidants and the risk of cardiovascular disease, total cancer, and all-cause mortality: a systematic review and dose-response meta-analysis of prospective studies. Am J Clin Nutr. (2018) 108:1069–91. 10.1093/ajcn/nqy097 30475962 PMC6250988

[12] Stepaniak U Micek A Grosso G Stefler D Topor-Madry R Kubinova R. Antioxidant vitamin intake and mortality in three central and eastern European urban populations: the HAPIEE study. Eur J Nutr. (2016) 55:547–60. 10.1007/s00394-015-0871-8 25762013 PMC4767874

[13] Bagheri A Naghshi S Sadeghi O Larijani B Esmaillzadeh A. Total, Dietary, and supplemental magnesium intakes and risk of all-cause, cardiovascular, and cancer mortality: a systematic review and dose-response meta-analysis of prospective cohort studies. Adv Nutr. (2021) 12:1196–210. 10.1093/advances/nmab001 33684200 PMC8321838

[14] Shi Z Chu A Zhen S Taylor AW Dai Y Riley M. Association between dietary zinc intake and mortality among Chinese adults: findings from 10-year follow-up in the Jiangsu Nutrition Study. Eur J Nutr. (2018) 57:2839–46. 10.1007/s00394-017-1551-7 29022177

[15] Said E Mousa S Fawzi M Sabry NA Farid S. Combined effect of high-dose vitamin A, vitamin E supplementation, and zinc on adult patients with diabetes: a randomized trial. J Adv Res. (2021) 28:27–33. 10.1016/j.jare.2020.06.013 33364042 PMC7753230

[16] Ashor AW Werner AD Lara J Willis ND Mathers JC Siervo M. Effects of vitamin C supplementation on glycaemic control: a systematic review and meta-analysis of randomised controlled trials. Eur J Clin Nutr. (2017) 71:1371–80. 10.1038/ejcn.2017.24 28294172

[17] Johnson CL Paulose-Ram R Ogden CL Carroll MD Kruszon-Moran D Dohrmann SM. National health and nutrition examination survey: analytic guidelines, 1999-2010. Vital Health Stat 2. (2013) (161):1–24. 25090154

[18] American Diabetes A. Diagnosis and classification of diabetes mellitus. Diabetes Care. (2013) 36 Suppl 1:S67–74. 10.2337/dc13-S067 23264425 PMC3537273

[19] Ahuja JK Moshfegh AJ Holden JM Harris E. USDA food and nutrient databases provide the infrastructure for food and nutrition research, policy, and practice. J Nutr. (2013) 143:241S−9S. 10.3945/jn.112.170043 23269654

[20] Kantor ED Rehm CD Du M White E Giovannucci EL. Trends in dietary supplement use among US adults from 1999-2012. JAMA. (2016) 316:1464–74. 10.1001/jama.2016.14403 27727382 PMC5540241

[21] U.S. Department of Health and Human Services; U.S. Department of Agriculture. 2015–2020 Dietary Guidelines for Americans. 8th ed. U.S. Department of Health and Human Services; U.S. Department of Agriculture: Washington, DC, USA, (2015). Available online at: http://health.gov/dietaryguidelines/2015/guidelines/ (accessed September 18, 2019).

[22] Tur JA Romaguera D Pons A. Does the diet of the Balearic population, a Mediterranean-type diet, ensure compliance with nutritional objectives for the Spanish population? Public Health Nutr. (2005) 8:275–83. 10.1079/PHN2004693 15918924

[23] Wright ME Mayne ST Stolzenberg-Solomon RZ Li Z Pietinen P Taylor PR. Development of a comprehensive dietary antioxidant index and application to lung cancer risk in a cohort of male smokers. Am J Epidemiol. (2004) 160:68–76. 10.1093/aje/kwh173 15229119

[24] Floegel A Kim DO Chung SJ Song WO Fernandez ML Bruno RS. Development and validation of an algorithm to establish a total antioxidant capacity database of the US diet. Int J Food Sci Nutr. (2010) 61:600–23. 10.3109/09637481003670816 20377495

[25] Ha K Kim K Sakaki JR Chun OK. Relative validity of dietary total antioxidant capacity for predicting all-cause mortality in comparison to diet quality indexes in US adults. Nutrients. (2020) 12:1210. 10.3390/nu12051210 32344879 PMC7282024

[26] Zhang YB Chen C Pan XF Guo J Li Y Franco OH. Associations of healthy lifestyle and socioeconomic status with mortality and incident cardiovascular disease: two prospective cohort studies. BMJ. (2021) 373:n604. 10.1136/bmj.n604 33853828 PMC8044922

[27] Wan Z Guo J Pan A Chen C Liu L Liu G. Association of serum 25-hydroxyvitamin D concentrations with all-cause and cause-specific mortality among individuals with diabetes. Diabetes Care. (2021) 44:350–7. 10.2337/dc20-1485 33168652

[28] Wolffenbuttel BHR Heiner-Fokkema MR Green R Gans ROB. Relationship between serum B12 concentrations and mortality: experience in NHANES. BMC Med. (2020) 18:307. 10.1186/s12916-020-01771-y 33032595 PMC7545540

[29] The Linkage of National Center for Health Statistics Survey Data to the National Death Index-2015 Linked Mortality File (LMF): Methodology Overview and Analytic Considerations (2019). Available online at: https://www.cdc.gov/nchs/data-linkage/mortality-public.htm (accessed April 11, 2019).

[30] Jayedi A Rashidy-Pour A Parohan M Zargar MS Shab-Bidar S. Dietary and circulating vitamin C, vitamin E, beta-carotene and risk of total cardiovascular mortality: a systematic review and dose-response meta-analysis of prospective observational studies. Public Health Nutr. (2019) 22:1872–87. 10.1017/S1368980018003725 30630552 PMC10260571

[31] Zhao LG Shu XO Li HL Gao J Han LH Wang J. Prospective cohort studies of dietary vitamin B6 intake and risk of cause-specific mortality. Clin Nutr. (2019) 38:1180–7. 10.1016/j.clnu.2018.04.016 29764693 PMC6551204

[32] Vischer UM Perrenoud L Genet C Ardigo S Registe-Rameau Y Herrmann FR. The high prevalence of malnutrition in elderly diabetic patients: implications for anti-diabetic drug treatments. Diabet Med. (2010) 27:918–24. 10.1111/j.1464-5491.2010.03047.x 20653750

[33] Forouhi NG Misra A Mohan V Taylor R Yancy W. Dietary and nutritional approaches for prevention and management of type 2 diabetes. BMJ. (2018) 361:k2234. 10.1136/bmj.k2234 29898883 PMC5998736

[34] Sun JW Shu XO Li HL Zhang W Gao J Zhao LG. Dietary selenium intake and mortality in two population-based cohort studies of 133 957 Chinese men and women. Public Health Nutr. (2016) 19:2991–8. 10.1017/S1368980016001130 27197889 PMC5063694

[35] Jayedi A Rashidy-Pour A Parohan M Zargar MS Shab-Bidar S. Dietary antioxidants, circulating antioxidant concentrations, total antioxidant capacity, and risk of all-cause mortality: a systematic review and dose-response meta-analysis of prospective observational studies. Adv Nutr. (2018) 9:701–16. 10.1093/advances/nmy040 30239557 PMC6247336

[36] Buijsse B Feskens EJ Kwape L Kok FJ Kromhout D. Both alpha- and beta-carotene, but not tocopherols and vitamin C, are inversely related to 15-year cardiovascular mortality in Dutch elderly men. J Nutr. (2008) 138:344–50. 10.1093/jn/138.2.344 18203902

[37] Genkinger JM Platz EA Hoffman SC Comstock GW Helzlsouer KJ. Fruit, vegetable, and antioxidant intake and all-cause, cancer, and cardiovascular disease mortality in a community-dwelling population in Washington County, Maryland. Am J Epidemiol. (2004) 160:1223–33. 10.1093/aje/kwh339 15583375

[38] Enstrom JE Kanim LE Klein MA. Vitamin C intake and mortality among a sample of the United States population. Epidemiology. (1992) 3:194–202. 10.1097/00001648-199205000-00003 1591317

[39] Otsuka R Tange C Nishita Y Kato Y Tomida M Imai T. Dietary diversity and all-cause and cause-specific mortality in Japanese community-dwelling older adults. Nutrients. (2020) 12:1052. 10.3390/nu12041052 32290256 PMC7230563

[40] Schwingshackl L Chaimani A Hoffmann G Schwedhelm C Boeing H. A network meta-analysis on the comparative efficacy of different dietary approaches on glycaemic control in patients with type 2 diabetes mellitus. Eur J Epidemiol. (2018) 33:157–70. 10.1007/s10654-017-0352-x 29302846 PMC5871653

[41] Liu G Guasch-Ferre M Hu Y Li Y Hu FB Rimm EB. Nut consumption in relation to cardiovascular disease incidence and mortality among patients with diabetes mellitus. Circ Res. (2019) 124:920–9. 10.1161/CIRCRESAHA.118.314316 30776978 PMC6417933

[42] Salganik RI. The benefits and hazards of antioxidants: controlling apoptosis and other protective mechanisms in cancer patients and the human population. J Am Coll Nutr. (2001) 20(Suppl. 5):464S−72S. discussion 73S−5S. 10.1080/07315724.2001.10719185 11603657

[43] El-Aal AA El-Ghffar EAA Ghali AA Zughbur MR Sirdah MM. The effect of vitamin C and/or E supplementations on type 2 diabetic adult males under metformin treatment: A single-blinded randomized controlled clinical trial. Diabetes Metab Syndr. (2018) 12:483–9. 10.1016/j.dsx.2018.03.013 29571976

[44] Jafarnejad S Mahboobi S McFarland LV Taghizadeh M Rahimi F. Meta-analysis: effects of zinc supplementation alone or with multi-nutrients, on glucose control and lipid levels in patients with type 2 diabetes. Prev Nutr Food Sci. (2019) 24:8–23. 10.3746/pnf.2019.24.1.8 31008092 PMC6456233

[45] Luu HN Wen W Li H Dai Q Yang G Cai Q. Are dietary antioxidant intake indices correlated to oxidative stress and inflammatory marker levels? Antioxid Redox Signal. (2015) 22:951–9. 10.1089/ars.2014.6212 25602689 PMC4376488

[46] Koya D Hayashi K Kitada M Kashiwagi A Kikkawa R Haneda M. Effects of antioxidants in diabetes-induced oxidative stress in the glomeruli of diabetic rats. J Am Soc Nephrol. (2003) 14(Suppl. 3):S250–3. 10.1097/01.ASN.0000077412.07578.44 12874441

[47] Fenercioglu AK Saler T Genc E Sabuncu H Altuntas Y. The effects of polyphenol-containing antioxidants on oxidative stress and lipid peroxidation in type 2 diabetes mellitus without complications. J Endocrinol Invest. (2010) 33:118-24. 10.1007/BF03346565. 10.1007/BF03346565 19834314

[48] Farvid MS Jalali M Siassi F Hosseini M. Comparison of the effects of vitamins and/or mineral supplementation on glomerular and tubular dysfunction in type 2 diabetes. Diabetes Care. (2005) 28:2458–64. 10.2337/diacare.28.10.2458 16186280

[49] Neri S Calvagno S Mauceri B Misseri M Tsami A Vecchio C. Effects of antioxidants on postprandial oxidative stress and endothelial dysfunction in subjects with impaired glucose tolerance and type 2 diabetes. Eur J Nutr. (2010) 49:409–16. 10.1007/s00394-010-0099-6 20213326

[50] Gutteridge JM. Free radicals in disease processes: a compilation of cause and consequence. Free Radic Res Commun. (1993) 19:141–58. 10.3109/10715769309111598 8244084

[51] Ghosh MK Mukhopadhyay M Chatterjee IB. NADPH-initiated cytochrome P450-dependent free iron-independent microsomal lipid peroxidation: specific prevention by ascorbic acid. Mol Cell Biochem. (1997) 166:35–44. 9046019 10.1023/a:1006841228483

[52] Palozza P. Prooxidant actions of carotenoids in biologic systems. Nutr Rev. (1998) 56:257–65. 10.1111/j.1753-4887.1998.tb01762.x 9763875

[53] Cutler RG Mattson MP. Measuring oxidative stress and interpreting its relevance in humans. In: Cutler RG Rodriguez H, editors. Oxidative Stress and Aging. New Jersey, NJ: World Scientific (2003). 10.1142/9789812775733_0008

[54] Pellegrini N Serafini M Salvatore S Del Rio D Bianchi M Brighenti F. Total antioxidant capacity of spices, dried fruits, nuts, pulses, cereals and sweets consumed in Italy assessed by three different in vitro assays. Mol Nutr Food Res. (2006) 50:1030–8. 10.1002/mnfr.200600067 17039458

[55] Pellegrini N Serafini M Colombi B Del Rio D Salvatore S Bianchi M. Total antioxidant capacity of plant foods, beverages and oils consumed in Italy assessed by three different in vitro assays. J Nutr. (2003) 133:2812–9. 10.1093/jn/133.9.2812 12949370

