# Supplementary material for: Dietary Antioxidant Indices in Relation to All-Cause and Cause-Specific Mortality Among Adults With Diabetes: A Prospective Cohort Study
Source: Front Nutr. 2022 May 4;9:849727. doi: 10.3389/fnut.2022.849727 (PMC9116439; doi:10.3389/fnut.2022.849727)
Supplement: Supplementary file 1 [file Data_Sheet_1.PDF]

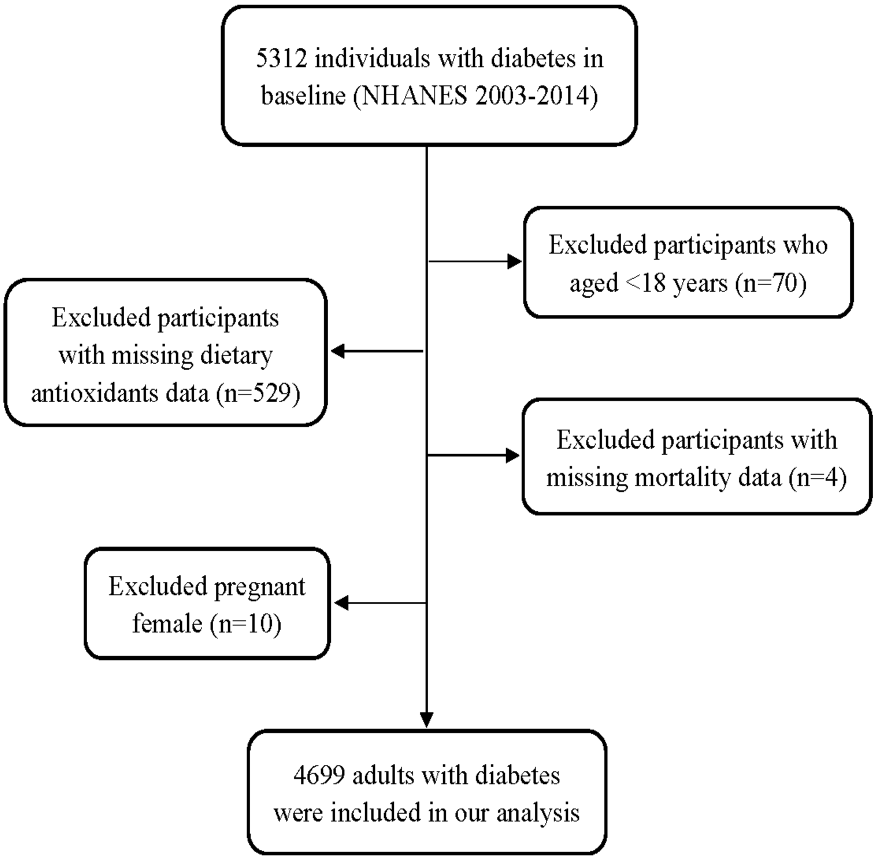

Supplementary Figure 1 Flow chat of participants included in the present study

Supplementary Table 1 Hazard Ratio (95% CIs) for all-cause and cause-specific mortality according to the tertiles of dietary antioxidant nutrients among adults with diabetes in National Health and Nutrition Examination Survey 2003–2014

Characteristics	Tertile 1	Tertile 2	Tertile 3	*P*-trend[1]
		Vitamin A		
All-cause mortality (Case/n)	298/1575	315/1562	300/1566	
Model 1	1.00	0.93(0.78-1.12)	0.70(0.57-0.87)	0.001
Model 2	1.00	0.98(0.82-1.19)	0.78(0.63-0.96)	0.015
Model 3	1.00	0.93(0.76-1.13)	0.78(0.63-0.97)	0.022
CVD mortality (Case/n)	72/1575	71/1562	72/1566	
Model 1	1.00	0.80(0.53-1.21)	0.61(0.39-0.97)	0.036
Model 2	1.00	0.86(0.57-1.13)	0.71(0.44-1.12)	0.135
Model 3	1.00	0.78(0.53-1.16)	0.69(0.45-1.04)	0.317
Cancer mortality (Case/n)	58/1575	59/1562	56/1566	
Model 1	1.00	0.85(0.53-1.37)	0.70(0.41-1.18)	0.167
Model 2	1.00	0.88(0.56-1.38)	0.76(0.45-1.30)	0.313
Model 3	1.00	0.89(0.55-1.43)	0.75(0.42-1.32)	0.305
		Vitamin C		
All-cause mortality (Case/n)	297/1590	305/1557	311/1556	
Model 1	1.00	0.86(0.69-1.07)	0.83(0.68-1.02)	0.087
Model 2	1.00	0.89(0.71-1.11)	0.90(0.73-1.11)	0.327
Model 3	1.00	0.91(0.73-1.14)	0.90(0.74-1.11)	0.341
CVD mortality (Case/n)	70/1520	70/1557	75/1556	
Model 1	1.00	0.82(0.55-1.21)	0.86(0.58-1.28)	0.483
Model 2	1.00	0.87(0.58-1.31)	0.96(0.64-1.43)	0.872
Model 3	1.00	0.92(0.60-1.41)	1.07(0.73-1.56)	0.745
Cancer mortality (Case/n)	59/1590	58/1557	56/1556	
Model 1	1.00	0.74(0.44-1.24)	0.63(0.40-0.99)	0.056
Model 2	1.00	0.77(0.46-1.31)	0.68(0.42-1.09)	0.119
Model 3	1.00	0.76(0.46-1.27)	0.66(0.41-1.06)	0.093
		Vitamin E		
All-cause mortality (Case/n)	434/1851	303/1636	176/1216	
Model 1	1.00	0.77(0.66-0.90)	0.67(0.56-0.81)	<0.001
Model 2	1.00	0.82(0.70-0.96)	0.74(0.61-0.89)	0.001
Model 3	1.00	0.81(0.69-0.96)	0.78(0.64-0.98)	0.014
CVD mortality (Case/n)	111/1740	67/1636	37/1216	
Model 1	1.00	0.68(0.50-0.93)	0.46(0.31-0.71)	0.001
Model 2	1.00	0.72(0.53-0.98)	0.52(0.33-0.80)	0.002
Model 3	1.00	0.71(0.52-0.98)	0.55(0.34-0.91)	0.011
Cancer mortality (Case/n)	76/1851	63/1636	34/1216	
Model 1	1.00	1.16(0.74-1.83)	0.92(0.54-1.57)	0.822
Model 2	1.00	1.20(0.76-1.90)	1.00(0.57-1.74)	0.935
Model 3	1.00	1.19(0.75-1.89)	0.91(0.50-1.66)	0.810
		Zinc		

All-cause mortality (Case/n)	394/1842	270/1429	249/1432	
Model 1	1.00	0.84(0.67-1.05)	0.85(0.66-1.08)	0.164
Model 2	100	0.87(0.70-1.08)	0.91(0.72-1.15)	0.404
Model 3	1.00	0.87(0.70-1.07)	0.95(0.75-1.19)	0.585
CVD mortality (Case/n)	94/1842	61/1429	60/1432	
Model 1	1.00	0.63(0.42-0.93)	0.63(0.41-0.96)	0.033
Model 2	1.00	0.64(0.43-0.95)	0.69(0.45-1.05)	0.080
Model 3	1.00	0.71(0.46-1.09)	0.62(0.41-0.93)	0.009
Cancer mortality (Case/n)	71/1842	51/1429	51/1432	
Model 1	1.00	0.70(0.46-1.07)	1.15(0.70-1.88)	0.578
Model 2	1.00	0.69(0.45-1.05)	1.20(0.74-1.96)	0.458
Model 3	1.00	0.66(0.43-1.02)	1.14(0.68-1.91)	0.600
		Magnesium		
All-cause mortality (Case/n)	401/1570	291/1570	221/1563	
Model 1	1.00	0.71(0.58-0.87)	0.56(0.45-0.69)	<0.001
Model 2	1.00	0.73(0.60-0.90)	0.61(0.48-0.76)	<0.001
Model 3	1.00	0.75(0.61-0.91)	0.65(0.52-0.81)	<0.001
CVD mortality (Case/n)	93/1570	66/1570	56/1563	
Model 1	1.00	0.66(0.47-0.92)	0.54(0.33-0.89)	0.014
Model 2	1.00	0.70(0.50-0.98)	0.61(0.37-1.01)	0.051
Model 3	1.00	0.71(0.52-0.98)	0.69(0.41-1.09)	0.093
Cancer mortality (Case/n)	77/1570	48/1570	48/1563	
Model 1	1.00	0.60(0.38-0.95)	0.79(0.47-1.34)	0.428
Model 2	1.00	0.61(0.40-0.95)	0.86(0.50-1.49)	0.611
Model 3	1.00	0.59(0.36-0.96)	0.80(0.45-1.40)	0.460
		Selenium		
All-cause mortality (Case/n)	385/1568	306/1573	222/1562	
Model 1	1.00	0.81(0.67-0.98)	0.70(0.57-0.88)	0.002
Model 2	1.00	0.84(0.69-1.01)	0.75(0.60-0.93)	0.008
Model 3	1.00	0.85(0.71-1.03)	0.79(0.62-0.98)	0.035
CVD mortality (Case/n)	88/1568	72/1573	55/1562	
Model 1	1.00	0.80(0.60-1.07)	0.57(0.36-0.91)	0.013
Model 2	1.00	0.83(0.62-1.11)	0.62(0.39-0.98)	0.031
Model 3	1.00	0.86(0.63-1.16)	0.67(0.40-0.99)	0.042
Cancer mortality (Case/n)	64/1568	57/1573	52/1562	
Model 1	1.00	0.92(0.52-1.64)	1.20(0.75-1.92)	0.443
Model 2	1.00	0.94(0.54-1.65)	1.23(0.76-1.99)	0.384
Model 3	1.00	0.88(0.51-1.52)	1.19(0.74-1.92)	0.464

CIs, Confidence intervals.

Model 1: adjusted for age, sex and race/ethnicity. Model 2: model 1 + body mass index, smoking status, drinking currently, education level, income level, exercise regularly. Model 3: model 2 + total energy intake, dietary supplements use, self-reported hypertension, dyslipidemia, heart disease, cancer, family history of diabetes, duration of diabetes and hemoglobin A_{1c}.

[1]Calculated by using the median value for each tertile of antioxidant nutrient as a continuous variable.

Supplementary Table 2 Hazard Ratio (95% CIs) for all-cause and cause-specific mortality according to dietary antioxidant quality score (DAQs) and dietary antioxidant index (DAI) among adults with diabetes in National Health and Nutrition Examination Survey 2003–2014, excluding adults with ≤ 2 years of follow-up.

	Dietary antioxidant quality score (DAQs)			*P*-trend[1]
	0-2	3-4	5-6	
All-cause mortality (Case/n)	259/1217	295/1745	173/11174	
Model 1	1.00	0.74(0.59-0.91)	0.66(0.55-0.80)	<0.001
Model 2	1.00	0.75(0.60-0.95)	0.70(0.56-0.88)	0.003
Model 3	1.00	0.71(0.56-0.90)	0.70(0.53-0.93)	0.012
CVD mortality (Case/n)	60/1217	74/1754	36/1174	
Model 1	1.00	0.72(0.46-1.13)	0.48(0.29-0.79)	0.004
Model 2	1.00	0.76(0.49-1.19)	0.55(0.34-0.89)	0.014
Model 3	1.00	0.70(0.45-1.08)	0.55(0.35-0.86)	0.011
Cancer mortality (Case/n)	46/1217	57/1754	30/1174	
Model 1	1.00	0.91(0.52-1.51)	0.88(0.52-1.48)	0.622
Model 2	1.00	0.96(0.59-1.57)	0.97(0.57-1.64)	0.908
Model 3	1.00	0.85(0.48-1.49)	0.71(0.39-1.28)	0.245
	Dietary antioxidant index (DAI)			
	Tertile 1	Tertile 2	Tertile 3	
All-cause mortality (Case/n)	288/1354	245/1402	194/1389	
Model 1	1.00	0.74(0.59-0.91)	0.66(0.55-0.80)	<0.001
Model 2	1.00	0.74(0.62-0.96)	0.74(0.60-0.89)	0.002
Model 3	1.00	0.77(0.63-0.95)	0.78(0.63-0.95)	0.019
CVD mortality (Case/n)	66/1354	64/1402	40/1389	
Model 1	1.00	0.78(0.52-1.16)	0.45(0.28-0.73)	0.001
Model 2	1.00	0.84(0.57-0.15)	0.52(0.33-0.83)	0.005
Model 3	1.00	0.80(0.54-1.17)	0.54(0.34-0.86)	0.007
Cancer mortality (Case/n)	49/1354	42/1402	42/1389	
Model 1	1.00	0.80(0.48-1.32)	1.02(0.63-1.67)	0.858
Model 2	1.00	0.82(0.50-1.34)	1.12(0.68-1.86)	0.606
Model 3	1.00	0.73(0.43-1.29)	0.84(0.40-1.79)	0.676

CIs, Confidence intervals.

Model 1: adjusted for age, sex and race/ethnicity.

Model 2: model 1 + body mass index, smoking status, drinking currently, education level, income level, exercise regularly.

Model 3: model 2 + total energy intake, dietary supplements use, self-reported hypertension, dyslipidemia, heart disease and cancer, family history of diabetes, duration of diabetes and hemoglobin A_{1c}.

[1]Calculated by using the median value for each DAQs or DAI category as a continuous variable.

Supplementary Table 3 Hazard Ratio (95% CIs) for all-cause and cause-specific mortality according to dietary antioxidant quality score (DAQs) and dietary antioxidant index (DAI) among adults with diabetes in National Health and Nutrition Examination Survey 2003–2014, excluding adults who took antioxidant supplements.

	Dietary antioxidant quality score (DAQs)			*P*-trend[1]
	0-2	3-4	5-6	
All-cause mortality				
Model 1	1.00	0.71(0.58-0.87)	0.65(0.52-0.80)	<0.001
Model 2	1.00	0.74(0.60-0.91)	0.71(0.57-0.88)	0.002
Model 3	1.00	0.70(0.57-0.87)	0.72(0.53-0.97)	0.021
CVD mortality				
Model 1	1.00	0.58(0.37-0.91)	0.48(0.29-0.80)	0.006
Model 2	1.00	0.61(0.39-0.97)	0.54(0.32-0.91)	0.020
Model 3	1.00	0.58(0.37-0.89)	0.54(0.33-0.89)	0.018
Cancer mortality				
Model 1	1.00	0.79(0.48-1.28)	0.88(0.51-1.51)	0.651
Model 2	1.00	0.83(0.51-1.34)	0.96(0.55-1.68)	0.891
Model 3	1.00	0.83(0.51-1.35)	0.94(0.53-1.67)	0.830
	Dietary antioxidant index (DAI)			
	Tretile 1	Tertile 2	Tertile 3	
All-cause mortality				
Model 1	1.00	0.71(0.57-0.89)	0.69(0.57-0.83)	<0.001
Model 2	1.00	0.76(0.61-0.95)	0.77(0.64-0.92)	0.005
Model 3	1.00	0.76(0.60-0.95)	0.80(0.65-0.99)	0.030
CVD mortality				
Model 1	1.00	0.66(0.46-0.96)	0.37(0.22-0.63)	<0.001
Model 2	1.00	0.73(0.50-1.06)	0.43(0.25-0.73)	0.001
Model 3	1.00	0.71(0.49-1.03)	0.44(0.26-0.75)	0.002
Cancer mortality				
Model 1	1.00	0.78(0.48-1.28)	1.21(0.73-1.98)	0.424
Model 2	1.00	0.83(0.51-.134)	1.33(0.79-1.15)	0.266
Model 3	1.00	0.79(0.49-1.28)	1.31(0.77-2.20)	0.301

CIs, Confidence intervals.

Model 1: adjusted for age and race/ethnicity.

Model 2: model 1 + body mass index, smoking status, drinking currently, education level, income level, exercise regularly.

Model 3: model 2 + total energy intake, self-reported hypertension, dyslipidemia, heart disease, cancer, family history of diabetes, duration of diabetes and hemoglobin A1c.

[1]Calculated by using the median value of DAQs and DAI in each group as a continuous variable.

Supplementary Table 4 Hazard Ratio (95% CIs) for all-cause and cause-specific mortality according to dietary antioxidant quality score (DAQs) and dietary antioxidant index (DAI) among adults with diabetes in National Health and Nutrition Examination Survey 2003–2015, further adjusting for dietary factors and dietary quality

	Dietary antioxidant quality score (DAQs)			*P*-trend
	0-2	3-4	5-6	
All-cause mortality				
Model 1	1.00	0.77(0.63-0.94)	0.70(0.53-0.92)	0.003
Model 2	1.00	0.76(0.61-0.95)	0.71(0.51-0.99)	0.035
Model 3	1.00	0.76(0.62-0.93)	0.68(0.51-0.91)	0.008
Model 4	1.00	0.80(0.65-0.98)	0.76(0.59-0.98)	0.025
CVD mortality				
Model 1	1.00	0.75(0.50-1.13)	0.56(0.35-0.90)	0.020
Model 2	1.00	0.80(0.53-1.21)	0.62(0.38-1.02)	0.063
Model 3	1.00	0.74(0.48-1.13)	0.55(0.30-1.01)	0.054
Model 4	1.00	0.76(0.50-1.16)	0.58(0.35-0.98)	0.043
Cancer mortality				
Model 1	1.00	0.72(0.45-1.17)	0.59(0.33-1.04)	0.062
Model 2	1.00	0.70(0.43-1.15)	0.58(0.32-1.04)	0.037
Model 3	1.00	0.75(0.51-1.09)	0.64(0.32-1.25)	0.186
Model 4	1.00	0.74(0.48-1.17)	0.62(0.32-1.21)	0.148
	Dietary antioxidant index (DAI)			
	Tertile 1	Tertile 2	Tertile 3	
All-cause mortality				
Model 1	1.00	0.76(0.63-0.92)	0.73(0.55-0.96)	0.014
Model 2	1.00	0.76(0.63-0.93)	0.72(0.55-0.96)	0.013
Model 3	1.00	0.76(0.63-0.91)	0.72(0.55-0.95)	0.009
Model 4	1.00	0.79(0.65-0.97)	0.78(0.63-0.98)	0.024
CVD mortality				
Model 1	1.00	0.74(0.51-1.06)	0.51(0.31-0.82)	0.005
Model 2	1.00	0.77(0.52-1.13)	0.58(0.31-0.99)	0.041
Model 3	1.00	0.70(0.47-1.06)	0.46(0.23-0.92)	0.026
Model 4	1.00	0.75(0.52-1.09)	0.52(0.32-0.85)	0.008
Cancer mortality				
Model 1	1.00	0.73(0.44-1.21)	0.73(0.35-1.53)	0.397
Model 2	1.00	0.70(0.42-1.15)	0.65(0.28-1.53)	0.308
Model 3	1.00	0.77(0.48-1.23)	0.80(0.35-1.82)	0.594
Model 4	1.00	0.76(0.47-1.23)	0.80(0.37-1.75)	0.558

CIs, Confidence intervals.

Model 1 : adjusted for age, sex, race/ethnicity, body mass index (BMI), smoking status, drinking currently, education level, income level, exercise regularly, total energy intake, dietary supplements use, self-reported hypertension, dyslipidemia, heart disease, cancer, family history of diabetes, duration of diabetes and hemoglobin A_{1c}.

Model 2: model 1 + monounsaturated fatty acids (MUFA)/saturated fatty acid (SFA) ratio,

polyunsaturated fatty acids (PUFAs)/saturated fatty acid (SFA) ratio, dietary cholesterol, and dietary fiber (in tertiles).
Model 3: model 1 + B vitamins intake including vitamin B_1, B_2, B_6 and folate (both in tertiles).
Model 4: model 1 + dietary quality indicated by health eating index (HEI-2015, in tertiles)

Supplementary Table 5 Hazard Ratio (95% CIs) for all-cause and cause-specific mortality according to dietary antioxidant quality score (DAQs) and dietary antioxidant index (DAI) among adults with diabetes in National Health and Nutrition Examination Survey 2003–2015, stratified by gender.

	Dietary antioxidant quality score (DAQs)			*P*-trend[1]	Dietary antioxidant quality score (DAQs)			*P*-trend[1]
	0-2	3-4	5-6		0-2	3-4	5-6	
	Male				Female			
All-cause mortality								
Model 1	1.00	0.73(0.56-0.96)	0.55(0.41-0.74)	<0.001	1.00	0.79(0.62-0.97)	0.67(0.49-0.91)	0.013
Model 2	1.00	0.78(0.60-1.03)	0.64(0.46-0.89)	0.006	1.00	0.80(0.64-1.01)	0.71(0.52-0.97)	0.033
Model 3	1.00	0.72(0.54-0.97)	0.58(0.38-0.88)	0.008	1.00	0.82(0.66-1.03)	0.72(0.53-0.97)	0.035
CVD mortality								
Model 1	1.00	0.72(0.44-1.18)	0.42(0.23-0.79)	0.005	1.00	0.84(0.47-1.48)	0.59(0.29-1.21)	0.138
Model 2	1.00	0.78(0.49-1.26)	0.52(0.27-0.99)	0.037	1.00	0.87(0.49-1.54)	0.62(0.31-1.27)	0.176
Model 3	1.00	0.74(0.45-1.20)	0.52(0.28-0.97)	0.034	1.00	0.89(0.49-1.62)	0.63(0.31-1.26)	0.167
Cancer mortality								
Model 1	1.00	0.89(0.52-1.54)	0.79(0.40-1.52)	0.463	1.00	0.59(0.27-1.21)	0.55(0.25-1.21)	0.161
Model 2	1.00	0.95(0.54-1.67)	0.90(0.43-1.86)	0.764	1.00	0.64(0.30-1.39)	0.59(0.27-1.30)	0.201
Model 3	1.00	0.86(0.44-1.67)	0.67(0.29-1.56)	0.343	1.00	0.63(0.32-1.24)	0.54(0.21-1.40)	0.206
	Dietary antioxidant index (DAI)				Dietary antioxidant index (DAI)			
	Tretile 1	Tertile 2	Tertile 3		Tretile 1	Tertile 2	Tertile 3	
All-cause mortality								
Model 1	1.00	0.69(0.51-0.93)	0.58(0.44-0.76)	<0.001	1.00	0.73(0.56-0.95)	0.68(0.50-0.93)	0.005
Model 2	1.00	0.76(0.56-1.04)	0.68(0.50-0.92)	0.016	1.00	0.75(0.58-0.97)	0.72(0.53-0.98)	0.016
Model 3	1.00	0.71(0.52-0.97)	0.60(0.39-0.91)	0.018	1.00	0.77(0.59-0.99)	0.71(0.52-0.97)	0.014
CVD mortality								
Model 1	1.00	0.82(0.54-1.25)	0.49(0.27-0.89)	0.014	1.00	0.59(0.32-1.08)	0.31(0.14-0.66)	0.003
Model 2	1.00	0.80(0.51-1.24)	0.50(0.27-0.91)	0.018	1.00	0.60(0.33-1.08)	0.32(0.15-0.69)	0.004

Model 3	1.00	0.80(0.51-1.26)	0.54(0.30-0.99)	0.041	1.00	0.61(0.34-1.10)	0.31(0.14-0.68)	0.003
Cancer mortality								
Model 1	1.00	0.85(0.47-1.53)	0.87(0.52-1.44)	0.663	1.00	0.70(0.32-1.51)	0.84(0.38-1.89)	0.552
Model 2	1.00	0.91(0.50-1.66)	0.98(0.54-1.78)	0.994	1.00	0.73(0.34-1.57)	0.87(0.39-1.96)	0.624
Model 3	1.00	0.77(0.40-1.51)	0.69(0.28-1.71)	0.446	1.00	0.82(0.39-1.76)	1.04(0.33-3.28)	0.969

CIs, Confidence intervals.

Model 1: adjusted for age and race/ethnicity.

Model 2: model 1 + body mass index, smoking status, drinking currently, education level, income level, exercise regularly.

Model 3: model 2 + total energy intake, dietary supplements use, self-reported hypertension, dyslipidemia, heart disease, cancer, family history of diabetes, duration of diabetes and hemoglobin A_{1c}.

[1]Calculated by using the median value for each DAQs and DAI category as a continuous variable.

Supplemental Table 6 The associations of dietary total antioxidant capacity (TAC) with mortality among people with diabetes.

	Dietary total antioxidant capacity (TAC)			*P*-trend
	Tertile 1	Tertile2	Tertile 3	
All-cause mortality				
Model 1	1.00	0.83(0.69-0.99)	0.69(0.56-0.84)	<0.001
Model 2	1.00	0.89(0.74-1.06)	0.76(0.62-0.93)	0.007
Model 3	1.00	0.87(0.73-1.04)	0.77(0.63-0.94)	0.009
CVD mortality				
Model 1	1.00	0.83(0.51-1.34)	0.58(0.38-0.88)	0.014
Model 2	1.00	0.85(0.51-1.40)	0.60(0.38-0.93)	0.026
Model 3	1.00	0.82(0.49-1.37)	0.62(0.40-0.97)	0.042
Cancer mortality				
Model 1	1.00	0.78(0.51-1.19)	0.64(0.42-0.98)	0.040
Model 2	1.00	0.81(0.52-1.26)	0.69(0.46-1.05)	0.083
Model 3	1.00	0.84(0.53-1.33)	0.69(0.44-1.06)	0.088

Model 1: adjusted for age, sex and race/ethnicity. Model 2: model 1 + body mass index, smoking status, drinking currently, education level, income level, exercise regularly. Model 3: model 2 + total energy intake, dietary supplements use, self-reported hypertension, dyslipidemia, heart disease, cancer, family history of diabetes, medication use for diabetes (insulin/diabetic pills/none), duration of diabetes and hemoglobin A1c.

Supplemental Table 7 The associations of dietary antioxidant quality score (DAQs) and dietary antioxidant index (DAI) with mortality among diabetics and non-diabetics.

	Dietary antioxidants quality score (DAQs)			*P*-trend	*P*-interaction
	0-2	3-4	5-6		
	All-cause mortality				
Diabetics	1.00	0.78(0.63-0.96)	0.67(0.50-0.88)	0.004	0.639
Non-diabetics	1.00	0.80(0.70-0.92)	0.64(0.55-0.75)	<0.001	
	CVD mortality				
Diabetics	1.00	0.72(0.47-1.09)	0.52(0.31-0.86)	0.012	0.035
Non-diabetics	1.00	0.80(0.59-1.09)	0.60(0.38-0.92)	0.018	
	Cancer mortality				
Diabetics	1.00	0.77(0.47-1.26)	0.56(0.29-1.06)	0.068	0.133
Non-diabetics	1.00	0.69(0.52-0.91)	0.68(0.49-0.96)	0.045	
	Dietary antioxidants index (DAI)				
	Tertile 1	Tertile 2	Tertile 2		
	All-cause mortality				
Diabetics	1.00	0.79(0.64-0.99)	0.67(0.51-0.90)	0.006	0.189
Non-diabetics	1.00	0.81(0.69-0.96)	0.62(0.51-0.75)	<0.001	
	CVD mortality				
Diabetics	1.00	0.72(0.48-1.07)	0.45(0.27-0.73)	0.001	0.006
Non-diabetics	1.00	0.97(0.70-1.35)	0.59(0.43-0.82)	0.002	
	Cancer mortality				
Diabetics	1.00	0.75(0.44-1.29)	0.66(0.30-1.43)	0.288	0.769
Non-diabetics	1.00	0.93(0.69-1.24)	0.68(0.48-0.95)	0.022	

Model was adjusted for age, sex, race/ethnicity, body mass index (BMI), smoking status, drinking currently, exercise, education level, income level, total energy intake, dietary supplements use, self-reported hypertension, dyslipidemia, heart disease, cancer, family history of diabetes and hemoglobin A_{1c}.

Supplementary Table 8 Hazard Ratio (95% CIs) for all-cause and CVD mortality according to dietary antioxidant quality score (DAQs) and dietary antioxidant index (DAI) among adults with diabetes in National Health and Nutrition Examination Survey 2003–2015, further adjusting for blood lipids, CRP, HOMA-IR and HOMA-β *

	Dietary antioxidant quality score (DAQs)			*P*-trend
	0-2	3-4	5-6	
All-cause mortality				
Model 1 (n=2223)	1.00	0.71(0.54-0.95)	0.61(0.40-0.92)	0.017
Model 2 (n=2945)	1.00	0.76(0.63-0.92)	0.75(0.56-0.99)	0.009
Model 3 (n=2299)	1.00	0.68(0.52-0.90)	0.58(0.39-0.89)	0.011
CVD mortality				
Model 1 (n=2223)	1.00	0.84(0.58-1.23)	0.59(0.38-0.91)	0.017
Model 2 (n=2945)	1.00	0.75(0.51-1.09)	0.59(0.37-1.03)	0.072
Model 3 (n=2299)	1.00	0.87(0.50-1.53)	0.61(0.33-1.11)	0.101
	Quality antioxidant index (DAI)			
	Tertil 1	Tertile 2	Tertile 3	
All-cause mortality				
Model 1 (n=2223)	1.00	0.64(0.48-0.86)	0.51(0.34-0.77)	0.002
Model 2 (n=2945)	1.00	0.81(0.61-1.09)	0.79(0.64-0.98)	0.082
Model 3 (n=2299)	1.00	0.62(0.46-0.83)	0.51(0.34-0.75)	0.001
CVD mortality				
Model 1 (n=2223)	1.00	0.66(0.36-1.22)	0.46(0.24-0.90)	0.021
Model 2 (n=2945)	1.00	0.80(0.57-1.12)	0.62(0.38-1.02)	0.065
Model 3 (n=2299)	1.00	0.72(0.39-1.33)	0.57(0.29-1.13)	0.112

CIs, confidence intervals; CVD, cardiovascular disease; CRP, C-reactive protein HOMA-IR, Homeostasis model assessment-insulin resistance;

Final model : adjusted for age, sex, race/ethnicity, body mass index (BMI), smoking status, drinking currently, education level, income level, exercise regularly, total energy intake, dietary supplements use, self-reported hypertension, dyslipidemia, heart disease, cancer, family history of diabetes, duration of diabetes and hemoglobin A_{1c}.

Model 1: final model + blood lipids including triglycerides, total cholesterol, HDL cholesterol and LDL cholesterol

Model 2: final model + CRP

Model 3: final model + HOMA-IR and HOMA-β

Supplementary Table 9. The differences of dietary antioxidants intake among people with diabetes and without diabetes.

	Diabetics	Non-diabetics	*P* value
Vitamin A	411.83(8.62)	432.43(1.76)	0.019
Vitamin C	79.58(1.07)	89.90(0.51)	<0.001
Vitamin E	6.77(0.06)	7.57(0.03)	<0.001
Zinc	10.59(0.10)	11.56(0.04)	<0.001
Selenium	102.99(0.72)	111.44(0.32)	<0.001
Magnesium	265.70(1.71)	288.06(0.79)	<0.001
